# PLIP: fully automated protein–ligand interaction profiler

**DOI:** 10.1093/nar/gkv315

**Published:** 2015-04-14

**Authors:** Sebastian Salentin, Sven Schreiber, V. Joachim Haupt, Melissa F. Adasme, Michael Schroeder

**Affiliations:** 1 Biotechnology Center (BIOTEC), TU Dresden, Tatzberg 47-49, 01307 Dresden, Germany; 2 Escuela de Ingeniería en Bioinformática, Universidad de Talca, Avda. Lircay s/n Talca, 3460000, Chile

## Abstract

The characterization of interactions in protein–ligand complexes is essential for research in structural bioinformatics, drug discovery and biology. However, comprehensive tools are not freely available to the research community. Here, we present the protein–ligand interaction profiler (PLIP), a novel web service for fully automated detection and visualization of relevant non-covalent protein–ligand contacts in 3D structures, freely available at projects.biotec.tu-dresden.de/plip-web. The input is either a Protein Data Bank structure, a protein or ligand name, or a custom protein–ligand complex (e.g. from docking). In contrast to other tools, the rule-based PLIP algorithm does not require any structure preparation. It returns a list of detected interactions on single atom level, covering seven interaction types (hydrogen bonds, hydrophobic contacts, pi-stacking, pi-cation interactions, salt bridges, water bridges and halogen bonds). PLIP stands out by offering publication-ready images, PyMOL session files to generate custom images and parsable result files to facilitate successive data processing. The full python source code is available for download on the website. PLIP's command-line mode allows for high-throughput interaction profiling.

## INTRODUCTION

**Figure 1.**
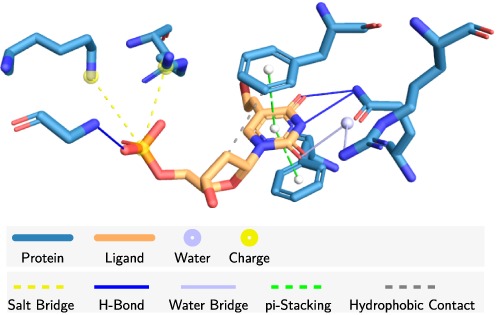
Example of interaction diagram generated with PLIP: *Varicella zoster* virus thymidine kinase (1OSN) binding the antiherpes drug brivudine-monophosphate. The binding is dominated by a double π-stacking and polar interactions at the terminal regions of the ligand.

The Protein Data Bank (PDB) (1) hosts nearly 100 000 deposited protein structures, with over 75% of them solved in complex with a small molecule ligand. Binding of a ligand to its host protein requires a specific arrangement of attractive, typically non-covalent contacts between both molecules. With such rich data at hand, we can gain deep insights into how ligands interact with their protein targets (2,3). Detailed characterization of these interaction patterns in individual cases is crucial to understand molecular recognition and protein function or to develop and optimize lead compounds. On the other hand, comparative high-throughput analyses of interaction patterns can considerably improve protein–ligand docking or virtual screening (4) and thus enhance *in silico* approaches in drug discovery. However, the scientific community lacks freely available tools to detect frequent non-covalent protein–ligand interactions such as hydrophobic interactions, hydrogen bonds, salt bridges and π-stacking (5). To this end, we herein present the free and open-source protein–ligand interaction profiler (PLIP), a fully automated and easy to use web server and command-line tool for protein–ligand interaction detection (Figure 1).

PLIP is complementary to other state-of-the-art web tools such as SwissDock (6), GalaxySite (7) or ProBiS (8) and can thus be applied in evaluation of docking results (Figure 4), drug design (Figure 5), binding site similarity assessment (3,9) and drug repositioning (10). The PLIP web service allows for comprehensive detection and visualization of protein–ligand interaction patterns from 3D structures, either directly from the PDB or in user-provided structures. Results for each binding site are provided as 3D interaction diagrams for manual inspection (online in JSmol and offline with PyMOL) as well as XML and flat text files for further processing.

All interactions are listed on atom-level detail, enabling analyses of specific binding characteristics. PLIP is freely accessible at projects.biotec.tu-dresden.de/plip-web without the need for registration or login. A short tutorial for new users as well as an extensive documentation is available on the website. The python source code is available for download on the PLIP website. Users interested in batch processing are encouraged to use the tool locally in command-line mode. A benchmark dataset of 30 literature-documented protein–ligand complexes is provided together with the source code.

## WEB SERVER DESCRIPTION

PLIP focuses on one-click processing of protein structures for the detection of interaction patterns. There are other tools, web pages and databases (11–20) as well as software from Chemical Computing Group (MOE), Accelrys and CLC bio available. Many of those tools, however, are commercial or can be used for visualization purposes only. Other offer only a limited selection of interaction types, require extensive preparation of input files or do not allow processing of custom structures. With PLIP, comprehensive interaction data for structures from PDB or external software is available without manual structure preparation and is made available as both diagrams and parsable result files.

### Input

The user needs to provide a protein–ligand complex in PDB format. Any structure from the RCSB PDB server (1) can be automatically loaded by providing a four-letter PDB ID or via free text search in protein and ligand names. Another option is to upload custom structures in PDB format (e.g. result files from docking or molecular dynamics software).

### Output

**Figure 2.**
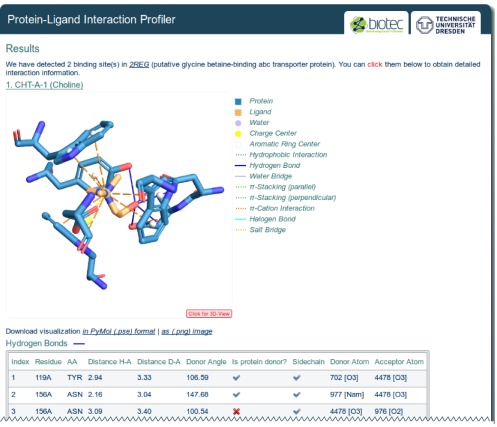
PLIP result page. An interaction diagram and a table with interaction data is provided for each binding site. JSMol applets allow to view the 3D interaction diagrams in the browser.

Figure 2 shows the result page for a typical analysis. For each binding site with a ligand, PLIP offers 2D and 3D interaction diagrams, a table with interaction details as well as downloadable result (XML and flat text) and visualization files (PNG and PyMOL session file). Details on interaction patterns can be accessed for each binding site by clicking on the identifier in the overview list. The results for each ligand are divided into a visualization section and a tabular listing of interaction data below (Figure 2). A JSMol-based 3D interaction diagram can be explored in the browser by clicking on the preview image. High-resolution images and PyMOL session files for preparation of custom publication-ready figures are available for download below the preview image. For manual inspection and successive processing, parsable XML or flat text files with interaction data are available at the bottom of the page.

## COMMAND-LINE TOOL DESCRIPTION

The python source code of PLIP is available as open-source software and allows to run computations locally. Additional to the features of the web server, the PLIP command-line tool offers advanced settings for output files and thresholds. It enables high-throughput computation of protein structures and can be readily integrated into analysis pipelines using the machine-readable result files. The usage and options are explained in the Supplementary Data.

## PLIP ALGORITHM

**Figure 3.**
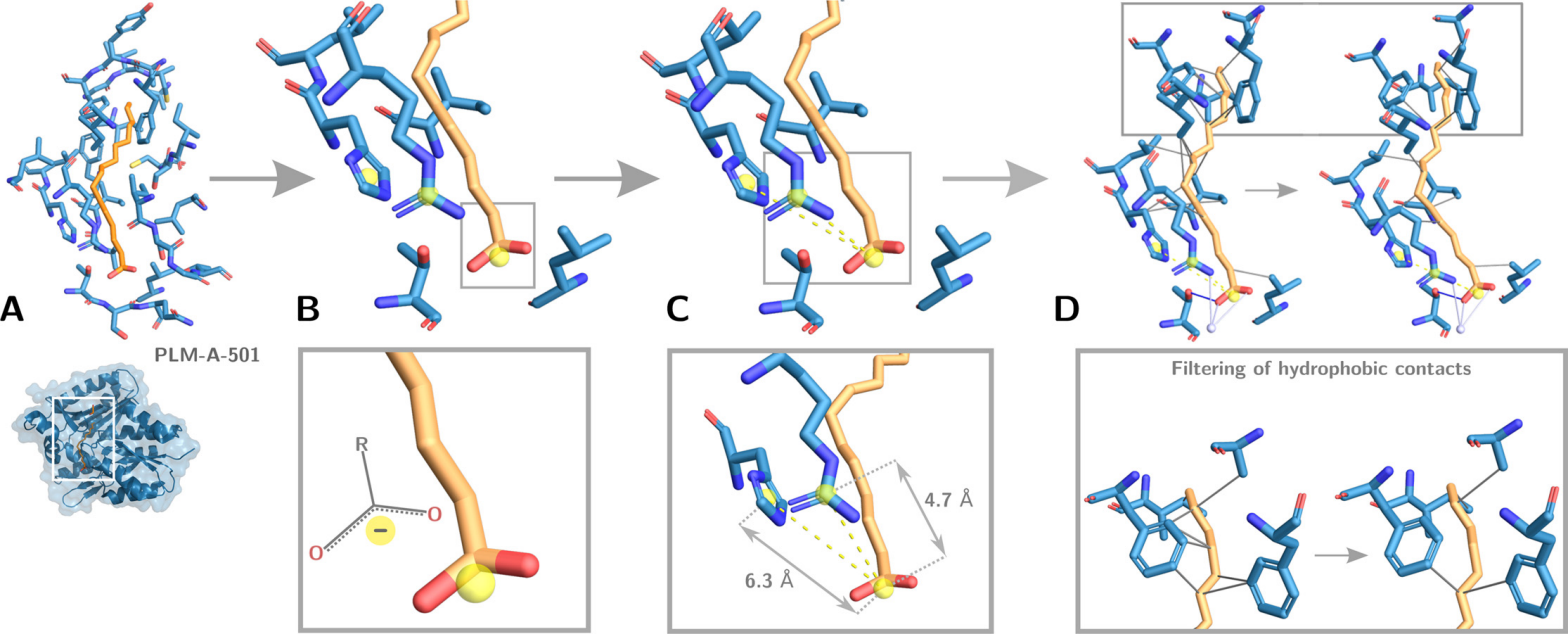
Example illustrating the four steps of PLIP in interaction detection for palmitic acid in *Bacillus subtilis* DegV protein (PDB ID 3FYS). (**A**) Structure preparation and detection of relevant ligands. (**B**) Functional characterization of molecules, here shown for the assignment of charges to amino acid side chains and the ligand carboxyl group. (**C**) Matching of interacting atoms using a rule-based system of geometric constraints. In the case of salt bridges, the distance between attracting charges is measured. (**D**) Filtering steps to minimize the number of depicted interactions, particularly important in the case of hydrophobic contacts (shown as solid gray lines).

PLIP uses four steps to detect and report relevant interactions: structure preparation, functional characterization, rule-based matching and filtering of interactions (Figure 3A–D). The analysis is exemplified by *Bacillus subtilis* DegV protein binding palmitic acid (PDB ID 3FYS).

In the preparation step, the input structure is hydrogenated and ligands extracted along with their binding sites. To this end, PLIP makes use of OpenBabel (21) for internal representation of molecules and most chemoinformatic calculations. In order to retain only specifically binding small molecules, PLIP uses a blacklist to exclude preparation artifacts, modified residues, ions and solvent compounds as ligands. The full blacklist is available for download on the PLIP website. In the example, only palmitic acid is kept as a relevant ligand (Figure 3A).

In order to find interacting groups (Figure 3B), the binding partners need to be functionally characterized first. This includes detection of hydrophobic atoms as well as acceptors/donors for hydrogen and halogen bonds. Furthermore, PLIP searches for aromatic rings and charge centers in protein and ligand. The latter functionalities are a precondition for formation of π-stacking, π-cation interactions or salt bridges. In the case of DegV with palmitic acid, charges can be assigned to two amino acids as well as the ligand carboxyl group (Figure 3B).

Following, putative interacting groups are matched by applying mostly geometric criteria (Figure 3C). Depending on the interaction type, this can include distance or angle constraints between arrangement of atoms. In the example case, the distances between atoms in positive and negative charges in the protein and palmitic acid are measured to decide whether to report a salt bridge (Figure 3C). The applied thresholds are taken from literature and are thus knowledge based. Most of them originate from analysis of large sets of high-quality protein structures in other studies. To account for low-quality structures and structural errors, some thresholds have been modified to be more permissive.

Last, filtering steps are used to eliminate redundant or overlapping interactions. As shown in Figure 3D this is especially important for hydrophobic contacts, which can be formed between any close apolar parts of ligand and protein. PLIP automatically searches for the most relevant contacts (shortest interatomic distance within the neighbourhood) to be reported. Some interaction types (e.g. salt bridges and hydrogen bonds) are very similar in their characteristics. In the case of detection of both interaction types for the same pairing of atoms, only one of them (e.g. a salt bridge) is reported.

Detailed descriptions of the algorithm and thresholds for each interaction type are available as Supplementary Data.

## VALIDATION

With the initial release of PLIP, we have included a test suite with 30 literature-validated examples (see Supplementary Data). They comprise diverse cases of protein–ligand complexes from PDB, covering all interaction types detectable by PLIP and resolutions from 1.2 to 3.3 Å. For each case, a test was implemented to check whether all interactions reported in the corresponding paper are being detected. The standard thresholds of PLIP have been carefully adapted to account for a broad range of interaction geometries while keeping the values as restrictive as possible. The test suite is available together with the source code on the PLIP website. Users are recommended to use these cases for testing when using custom thresholds and encouraged to contribute additional examples.

## EXAMPLES

PLIP can be used for both—structures from the PDB archive and structure files from other tools. It is therefore possible to integrate PLIP into pipelines for analyses related to protein–ligand binding, e.g. post processing of docking results or inhibitor design.

### Example 1: docking post processing

**Figure 4.**
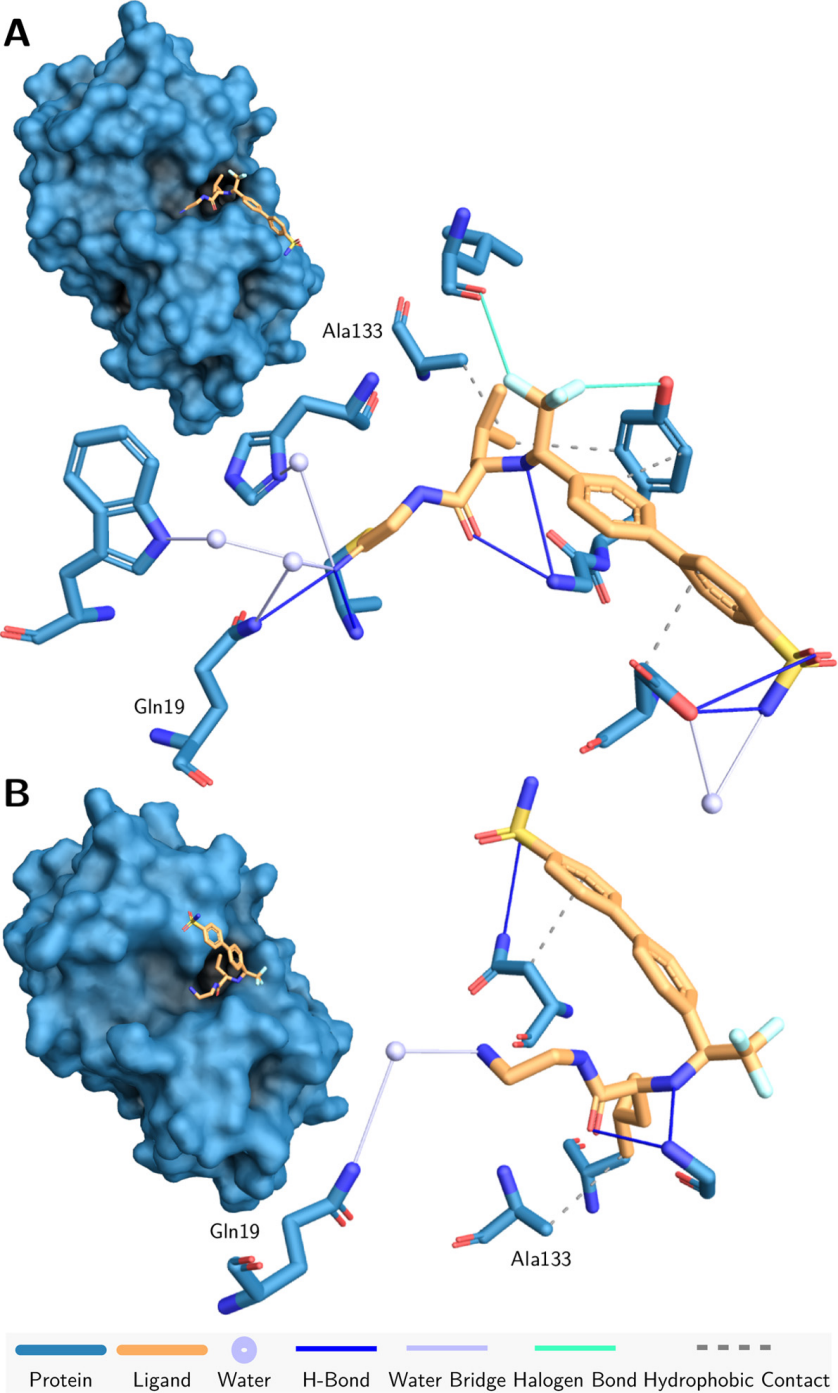
Evaluating docking results with PLIP. Natural (**A**) and alternative pose from redocking (**B**) of Cathepsin K with a small molecule inhibitor (PDB ID 1VSN). Shared interacting residues are labeled. The second pose lacks characteristic halogen bonds.

The elimination of false positive results from docking results can be performed using post processing pipelines (22). One approach is to use existing knowledge on key interactions with the protein of interest in order to filter from high-scoring poses. Cathepsin K in complex with a small molecule inhibitor (PDB ID 1VSN) was used for a redocking experiment using the SwissDock server at swissdock.ch. While the top prediction corresponds to the pose found in the crystal structure, the first alternative pose shows a clearly different ligand conformation, but comparable SwissDock fitness scores. PLIP was used to analyze the interaction patterns in the complex from the crystal structure (Figure 4A) and the complex with the alternative pose (Figure 4B) from docking.

In the alternative pose, the ligand part containing the aromatic rings is flipped to the opposite direction. A rich network of hydrogen bonds and water bridges can only be observed for the correct pose (Figure 4A). Most strikingly, however, the characteristic halogen bonds are completely missing in the alternative pose, leaving the trifluoride group exposed. With the detailed interaction patterns at hand, it is thus possible to identify wrong poses based on previous knowledge.

### Example 2: inhibitor design

**Figure 5.**
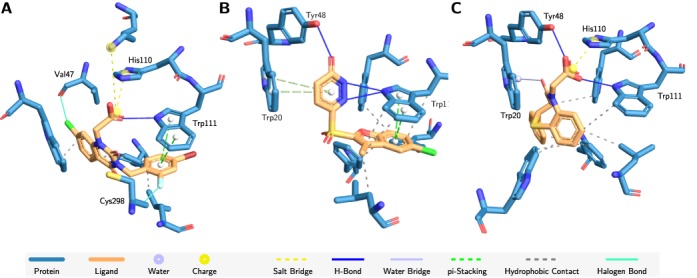
Human aldose reductase with different inhibitors. (**A**) Zenarestat (1IEI), (**B**) a sulfonyl-pyridazone inhibitor (1Z89) and (**C**) a benzothiazepine inhibitor (3P2V). While the first and the last share a salt bridge to His110 and the H-Bonds to Tyr48 and Trp111, there is a common π-stacking to Trp111 in the first two. Unique interactions are, among others, two halogen bonds in zenarestat to the backbones of Val47 and Cys298, additional stacking with Trp20 for the benzothiazepine inhibitor and a water bridge to Trp20 in the last inhibitor. Large parts of all ligands bind via hydrophobic contacts.

In initial stages of inhibitor design or prior to library screening, comparative analyses of known binding patterns with the target protein help identifying key residues. Here, PLIP is used to analyse interactions in three complexes of different inhibitors (PDB IDs 1IEI, 1Z89, 3P2V) with human aldose reductase (Figure 5).

Aldose reductase binds ligands via induced fit, leading to drastic conformational changes around the binding pocket (23). The three considered inhibitors show common interaction patterns but also individual subpatterns. While both—zenarastat (Figure 5A) and the benzothiazepine inhibitor (Figure 5C)—form a salt bridge to His110 and a hydrogen bond to Trp111 via their carboxyl groups, the interaction pattern of the sulfonyl-pyridazone inhibitor (Figure 5B) lacks this interaction. Without the carboxyl group only one hydrogen bond to Tyr48 is formed. This interaction can also be observed in complex with the benzothiazepine inhibitor.

Although all inhibitors have aromatic rings, only two form π-stacking interactions with Trp111. One of the most unique interaction patterns can be seen in the complex with zenarestat, where halogen bonds to the protein backbone are formed from both ends of the inhibitor.

## CONCLUSION

PLIP is the first web service to provide comprehensive analysis and visualization of non-covalent protein–ligand interactions with one-click loading of structures. With the availability of PyMOL session files and results in parsable formats, both—manual inspection and computational processing of interaction data—are possible. Furthermore, the availability of PLIP source code enables local batch processing, customization of the algorithm for special applications as well as active development of the tool in the community.

## SUPPLEMENTARY DATA

 Supplementary Data are available at NAR online.

SUPPLEMENTARY DATA
