# Supplementary material for: PLIP: fully automated protein–ligand interaction profiler
Source: Nucleic Acids Res. 2015 Apr 14;43(Web Server issue):W443–7. doi: 10.1093/nar/gkv315 (PMC4489249; doi:10.1093/nar/gkv315)
Supplement: SUPPLEMENTARY DATA [file supp_43_W1_W443__index.html]

PLIP: fully automated protein–ligand interaction profiler — PLIP: fully automated protein–ligand interaction profiler — SUPPLEMENTARY DATA 

# PLIP: fully automated protein–ligand interaction profiler

## SUPPLEMENTARY DATA

**Files in this Data Supplement:**

- SUPPLEMENTARY DATA
