# Supplementary material for: PLIP: fully automated protein–ligand interaction profiler
Source: Nucleic Acids Res. 2015 Apr 14;43(Web Server issue):W443–7. doi: 10.1093/nar/gkv315 (PMC4489249; doi:10.1093/nar/gkv315)
Supplement: SUPPLEMENTARY DATA [file supp_gkv315_nar-00254-web-b-2015-File003.pdf]

# 1 PLIP Command Line Tool

## 1.1 Setup

PLIP is written in Python and has the following dependencies (python packages) to run properly:

- lxml
- openbabel $\geq$ 2.3.2
- pybel
- numpy
- pymol $\geq$ 1.7

Imagemagick is optional for PLIP, but is used to scale the output images.

## 1.2 Input and Options

The program can be used on any PDB file from the PDB archive (online) or output files from other tools in PDB format without further preparation. To analyze a structure from your machine, run

```
python plip-cmd.py -f <file.pdb>
```

To fetch and analyze a structure from the PDB archive (requires internet connection), run PLIP using

```
python plip-cmd.py -i <4-letter-PDB-ID>
```

Several options are available, which are shown by calling the help message with

```
python plip-cmd.py -h
```

| | |
|---|---|
| `-h, -help` | Shows help message |
| `-f INPUT, -file INPUT` | Read in a file in PDB format |
| `-i PDBID, -input PDBID` | Fetch and analyze a file from the online PDB archive |
| `-o OUTPATH, -out OUTPATH` | Sets the output folder for PLIP result files (standard: working directory) |
| `-v, -verbose` | Sets verbose mode |
| `-p, -pics` | Generate ray-traced pictures |
| `-x, -xml` | Generate XML format reports |
| `-y, -pymol` | Generate PyMOL session files |
| `-maxthreads` | Max. number of simultaneously processed binding sites |

Setting maxthreads to 0 will deactivate multiprocessing. By default, parallel processing is turned off for usage on Windows.

# 2 Validation

For the validation of the PLIP algorithm we composed a set of 30 diverse protein-ligand complexes with non-covalent interactions described in literature. The validation cases (Table 3) cover all interaction types detectable with PLIP and structures with different resolutions (1.2 to 3.3 Å). To implement the tests, the set of interacting residues for a specific interaction type was compared against the set PLIP detects for the given complex. Contact both reported in literature and detected with PLIP are listed as *true positives.* Additional contacts detected by PLIP, but not listed in the publication are labeled as *false positives.* During testing, initial thresholds from literature have been modified (in the case of distance thresholds max. ± 1 Å) in order to prevent false negative results. False negative contacts still listed in Table 3 have been checked manually. They either result from errors in the publication, different interpretation of contact (as often the case for types with similar characteristics as salt bridges and hydrogen bonds), or unreasonable thresholds.

# 3 PLIP Algorithm

PLIP uses a rule-based system for detection of non-covalent interactions between protein residues and ligands. Information on chemical groups able to participate in a specific interaction (e.g. requirements for hydrogen bond donors) and interaction geometry (e.g. distance and angle thresholds) from literature are used to detect characteristics of non-covalent interactions between contacting atoms of protein and ligands. For each binding site, the algorithm searches first for atoms or atom groups in the protein and ligand which could possibly be partner in specific interactions. Subsequently, geometric rules are applied to match groups in protein and ligand forming an interaction.

Previous to the detection step for the interactions, PLIP extracts all ligands contained in the structure. Ions, DNA, modified amino acids, preparation artifact and solvent components are blacklisted as ligands. The complete list used for the filtering step is available on our website. Additionally, we use the BioLiP ([1]) list of possible artifacts to remove ligands which are in this list and appear 10 times or more in a structure.

# 4 Detection of functional groups, atoms or molecules

## 4.1 Binding site atoms

The binding site distance cutoff is determined by adding up `BS_DIST_MAX` to the maximum extent of the ligand (maximum distance of a ligand atom to ligand centroid). All protein atoms within this distance cutoff to any binding site atoms are counted as belonging to the binding site.

## 4.2 Hydrophobic Atoms

An atom is classified as *hydrophobic* if it is a carbon and has only carbon or hydrogen atoms as neighbours.

## 4.3 Aromatic Rings

OpenBabel is used to identify rings (SSSR perception) and their aromaticity. In cases where no aromaticity is reported by OpenBabel, the ring is checked for planarity. To this end, the normals of each atom in the ring to its neighbors is calculated. The angle between each pair of normals has to be less than `AROMATIC_PLANARITY`. If this holds true, the ring is also considered as aromatic.

## 4.4 Hydrogen Bond Donors and Acceptors

OpenBabel [2] is used to identify hydrogen bond donor and acceptor atoms. Halogen atoms are excluded as hydrogen bond acceptors.

## 4.5 Charged Groups

The detection of charged groups is only exhaustive for the binding site, not the ligand. For proteins, positive charges are attributed to the side chain nitrogens of Arginine, Histidine and Lysine. Negative charged are assigned to the carboxyl groups in Aspartic Acid and Glutamic Acid.

In ligands, positive charges are assigned to quaterny ammonium groups, tertiary amines (assuming the nitrogen could pick up a hydrogen and thus get charged), sulfonium and guanidine groups. Negative charges are reported for phosphate, sulfonate, sulfonic acid and carboxylate.

## 4.6 Halogen bond donors and acceptors

Assuming that halogen atoms are not present in proteins (unless they are artificially modified), halogen bond donors are searched for only in ligands. All fluorine, chlorine, bromide or iodine atoms connected to a carbon atom qualify as donors. Halogen bond acceptors in proteins are all proximal oxygen, nitrogen or sulfur atoms connected to carbon, nitrogen, phosphor or sulfur atoms.

## 4.7 Water

Water atoms are assigned to a ligand-binding site complex if their oxygen atoms are within a certain cutoff to the ligand. The cutoff is determined by adding up `BS_DIST_MAX` value to the maximum extent to the ligand (maximum distance of a ligand atom to ligand centroid). This means the farthest distance of a ligand to a water atom is `BS_DIST_MAX`.

# 5 Detection of Interactions

For an overview on geometric cutoffs used for the prediction of interactions, see Table 1.

## 5.1 Hydrophobic Interactions

### 5.1.1 Detection Step

As hydrophobic interactions result from entropic changes rather than attractive forces between atoms, there are no clear geometries of hydrophobic association. The observed attraction

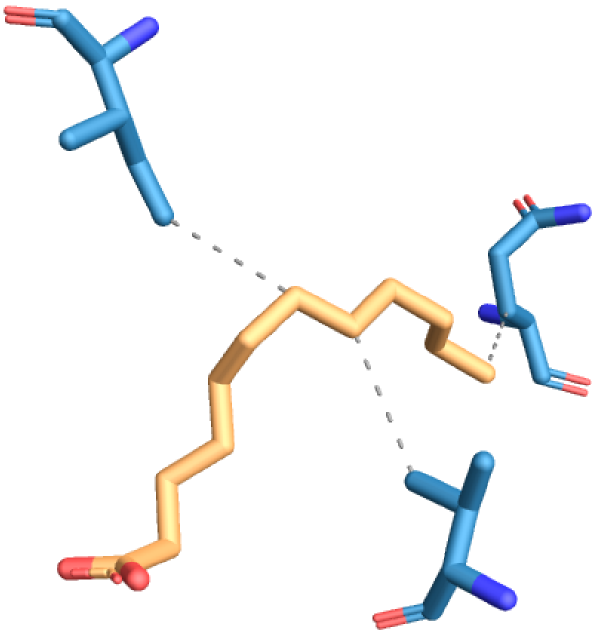

Figure 1: Hydrophobic interactions between HNF4 alpha ligand binding domain and lauric acid (1M7W:DAO-A-700)

between hydrophobic atoms decays exponentially with the distance between them [3]. A generous cutoff was chosen, identifying a prime set of hydrophobic interactions between all pairs of hydrophobic atoms within a distance of `HYDROPH_DIST_MAX`.

### 5.1.2 Reduction Step

Since the number of hydrophobic interactions with such an one-step approach can easily surpass all other interaction types combined, it may strongly influence subsequent evaluation or interaction fingerprinting. To overcome this problem, the number of hydrophobic interactions is reduced in several steps. First, hydrophobic interactions between rings interacting via $\pi$-stacking are removed. This is done because stacking already involves hydrophobic interactions [4]. Second, two clustering steps are applied. If a ligand atom interacts with several binding site atoms in the same residue, only the interaction with the closest distance is kept. Subsequently, the set of hydrophobic interactions is checked from the opposite perspective: if a protein atom interacts with several neighboring ligand atoms, just the interaction with the closest distance is kept. Together, these reduction steps help to report only the most representative hydrophobic interactions.

## 5.2 Hydrogen Bonds

### 5.2.1 Detection Step

A hydrogen bond between a hydrogen bond donor and acceptor (subsection 4.4) is reported if several geometric requirements are fulfilled. The distance has to be less than `HBOND_DIST_MAX` and the angle at the donor group (D-H...A) above `HBOND_DON_ANGLE_MIN`.

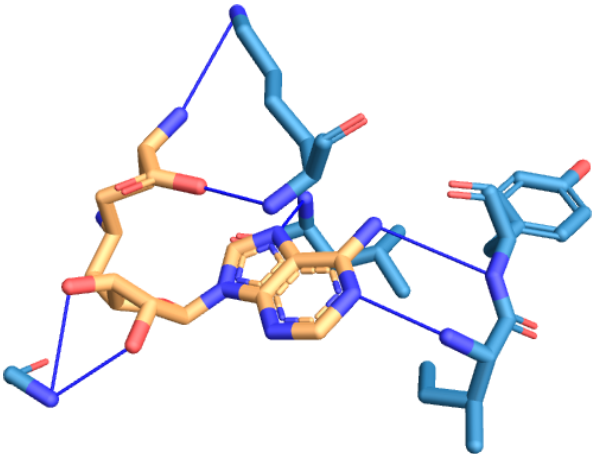

Figure 2: Hydrogen bonds between *M. jannaschii* Nep1 and Sinefungin (3BBH:SFG-A-206)

#### 5.2.2 Reduction Step

Since salt bridges involve purely electrostatic interactions as well as hydrogen bonds [5], it is not meaningful to report both interaction types between the same groups. Thus, hydrogen bonds between atoms are removed if they belong to groups that already form a salt bridge to that atom. As a general rule, a hydrogen bond donor can take part in only one hydrogen bond, while acceptor atoms can be partners in multiple hydrogen bonds (e.g. bifurcated hydrogen bonds). For multiple possible hydrogen bonds from one donor, only the contact with the donor angle closer to 180 °is kept.

### 5.3 Aromatic stacking

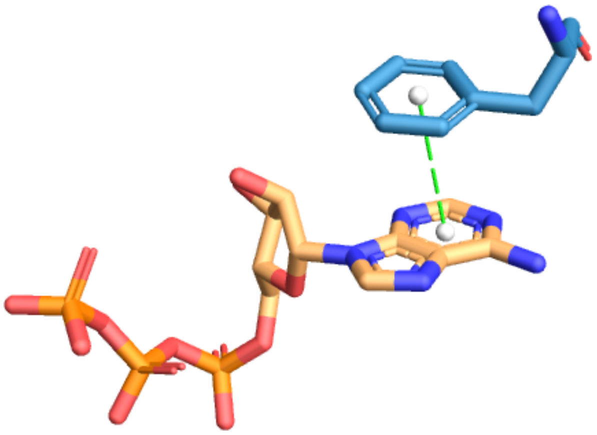

Figure 3: Aromatic stacking between *Trypanosoma brucei* RNA editing ligase 1 and ATP (1XDN:ATP-A-501)

$\pi$-stacking for two aromatic rings is reported whenever their centers are within a distance of `PISTACK_DIST_MAX` [6], the angle deviates no more than `PISTACK_ANG_DEV` from the optimal angle of 90° for T-stacking or 180° for P-stacking. Additionally, each ring center is projected

onto the opposite ring plane. The distance between the other ring center and the projected point (i.e. the offset) has to be less than `PISTACK_OFFSET_MAX`. This value corresponds approximately to the radius of benzene + 0.5 Å.

### 5.4 Pi-cation interactions

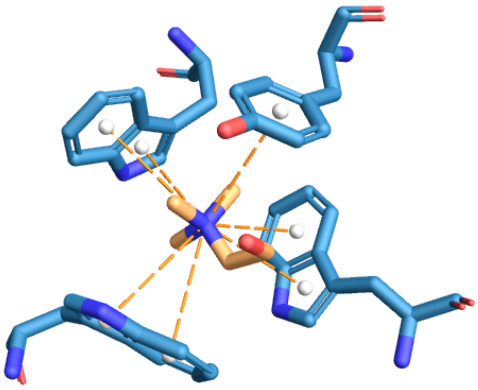

Figure 4: Pi-cation interactions between ABC-transporter choline binding protein and choline (2REG:CHT-A-1)

$\pi$-cation interactions are reported for each pairing of a positive charge and an aromatic ring if the distance between the charge center and the aromatic ring center is less than `PICATION_DIST_MAX` [7]. In the case of a putative $\pi$-cation interaction with a tertiary amine of the ligand, an additional angle criterion is applied (see documentation in the source code).

### 5.5 Salt Bridges

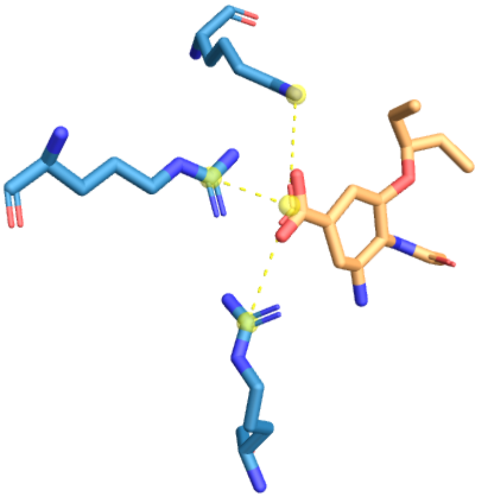

Figure 5: Salt bridges between Shanghai N9 neuraminidase and oseltamivir carboxylate (4MWW:G39-A-513)

Whenever two centers of opposite charges come within a distance of `SALTBRIDGE_DIST_MAX`,

a salt bridge is reported [8]. In contrast to hydrogen bonds, there are no additonal geometric restrictions.

## 5.6 Water-bridged hydrogen bonds

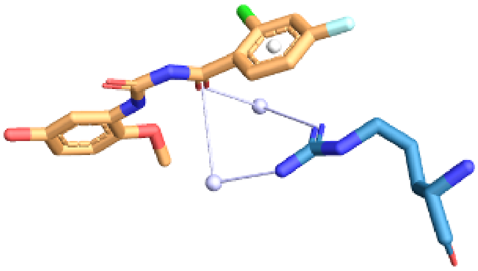

Figure 6: Water-bridged hydrogen bonds between human liver glycogen phosphorylase and acyl urea inhibitor (2ATI:IHU-A-848)

### 5.6.1 Detection Step

While residues can be bridged by more than one water molecule, for the prediction in this script the only case considered is one water molecule bridging ligand and protein atoms via hydrogen bonding. The water molecule has to be positioned between hydrogen bond donor/acceptor pairs of ligand and protein with distances of the water oxygen within `WATER_BRIDGE_MINDIST` and `WATER_BRIDGE_MAXDIST` to the corresponding polar atoms of the donor or acceptor groups. If a constellation with a water atom fulfills these requirements, two angles are checked. The angle $\omega$ between the acceptor atom, the water oxygen and donor hydrogen has to be within `WATER_BRIDGE_OMEGA_MIN` and `WATER_BRIDGE_OMEGA_MAX`. Additionally, the angle $\theta$ between the water oxygen, the donor hydrogen and the donor atom has to be larger than `WATER_BRIDGE_THETA_MIN`. The geometric constraints have been taken from [9].

### 5.6.2 Reduction Step

Similar to standard hydrogen bonds, a water molecule is only allowed to participate as donor in two hydrogen bonds (two hydrogen atoms as donors). In the case of more than two possible hydrogen bonds for a water molecule as donor, only the two contacts with a water angle closest to 110 °are kept.

## 5.7 Halogen bonds

Halogen bonds are reported for each pairing of halogen bond acceptor and donor group having a distance of less than `HALOGEN_DIST_MAX` and angles at the donor and acceptor group of `HALOGEN_DON_ANGLE` and `HALOGEN_ACC_ANGLE` with a deviation of no more than `HALOGEN_ANG_DEV`.

## 5.8 Currently non-supported interaction types

- Metal coordination

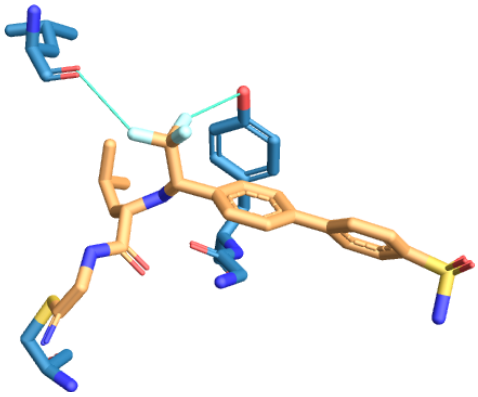

Figure 7: Halogen bonds between Cathepsin K and an inhibitor (1VSN:NFT-A-238)

- Covalent bonds
- Weak hydrogen bonds involving carbon atoms
- Halogen-Water-Hydrogen Bridges
- Water bridges of higher degree (bridging over more than one water molecule)

# 6 Appendix

Table 1: **Standard angle and distance thresholds** used for prediction. Angles in degree and distances in Ångström.

| Variable | Value | Description | Ref. |
|---|---|---|---|
| BS_DIST_MAX | 8.5 Å | Max. Cutoff for determination of binding site atoms | - |
| AROMATIC_PLANARITY | 7.5 ° | Max. Cutoff for planarity criterion in aromatic ring detection | - |
| HYDROPH_DIST_MAX | 4.0 Å | Max. distance of carbon atoms for a hydrophobic interaction | - |
| HBOND_DIST_MAX | 4.1 Å | Max. distance between acceptor and donor in hydrogens bonds | [10] |
| HBOND_DON_ANGLE_MIN | 100 ° | Min. angle at the hydrogen bond donor (D-H...A) | [10] |
| PISTACK_DIST_MAX | 7.5 Å | Max. distance between ring centers for stacking | [6] |
| PISTACK_ANG_DEV | 30 ° | Max. deviation from optimum angle for stacking | - |
| PISTACK_OFFSET_MAX | 2.0 Å | Max. offset between aromatic ring centers for stacking | - |
| PICATION_DIST_MAX | 6.0 Å | Max. distance between charge and aromatic ring centers | [7] |
| SALTBRIDGE_DIST_MAX | 5.5 Å | Distance between two centers of charges in saltbridges | [8] |
| HALOGEN_DIST_MAX | 4.0 Å | Max. distance between oxygen and halogen | [11] |
| HALOGEN_ACC_ANGLE | 120 ° | Optimal halogen bond acceptor angle | [11] |
| HALOGEN_DON_ANGLE | 165 ° | Optimal halogen bond donor angle | [11] |
| HALOGEN_ANGLE_DEV | 30 ° | Max. deviation from optimal halogen bond angles | [11] |
| WATER_BRIDGE_MINDIST | 2.5 Å | Min. distance between water oxygen and polar atom | [9] |
| WATER_BRIDGE_MAXDIST | 4.0 Å | Max. distance between water oxygen and polar atom | [9] |
| WATER_BRIDGE_OMEGA_MIN | 75 ° | Min. angle between acceptor, water oxygen and donor hydrogen | [9] |
| WATER_BRIDGE_OMEGA_MAX | 140 ° | Max. angle between acceptor, water oxygen and donor hydrogen | [9] |
| WATER_BRIDGE_THETA_MIN | 100 ° | Min. angle between water oxygen, donor atom and hydrogen | [9] |

Table 2: Abbreviations used in the textual report files and their explanation.

| **Abbreviation** | **Explanation** |
|---|---|
| RESNR | Residue number (as in the PDB file) |
| RESTYPE | Amino acid type |
| RESCHAIN | Chain of interacting residue |
| DIST | Distance between interacting atoms |
| DIST_H-A | Distance between hydrogen and acceptor atoms |
| DIST_D-A | Distance between donor and acceptor atoms |
| DIST_A-W | Distance between acceptor and water oxygen atoms |
| DIST_D-W | Distance between donor and water oxygen atoms |
| LIGCARBONIDX | Atom ID of ligand carbon atom |
| PROTCARBONIDX | Atom ID of protein carbon atom |
| ITYPE | Identifier for interaction type |
| PROTISDON | True if protein is has the donor group for the interaction |
| DONORIDX | Atom ID of donor atom |
| ACCEPTORIDX | Atom ID of acceptor atom |
| DON_ANGLE | Angle at the donor group |
| WATER_ANGLE | Angle between water oxygen, donor and acceptor group |
| WATER_IDX | Atom ID of water atom |
| PROTISPOS | True if protein provides a positive charge for the interaction |
| LIG_GROUP | Ligand functional group |
| LIG_IDX_LIST | List of atom IDs from participating atoms |
| OFFSET | Offset from aromatic ring |
| PROTCHARGED | True if the protein provides a charge for the interaction |
| ACC_ANGLE | Angle at the acceptor group |
| DONORTYPE | Type of donor atom |
| ACC_IDX | Atom ID of acceptor atom |
| SIDECHAIN | True if the hydrogen bond is formed with an amino acid sidechain |
| LIGCOO | Coordinates of ligand interacting atom in the PDB file |
| PROTCOO | Coordinates of protein interacting atom in the PDB file |

Table 3: Validation set for PLIP. Listing **HAL** Halogen Bonds, **HBO** H-Bonds, **HPI** Hydrophobic Interactions, **PCT** $\pi$-Cation interactions, **PST** $\pi$-Stacking, **SBR** Salt Bridges, **WAT** Water Bridges. True positives are interactions reported by PLIP and in literature. False negatives are interactions not detected by PLIP, false positives are not listed in the publication.

| PDB ID | Binding Site | Ref. | TP | FP | FN |
|---|---|---|---|---|---|
| 1AGL | P84-A-400 | [12] | **HAL**:L145 **HPI**:T150 **WAT**:V147 | | |
| 1AJC | THA-A-999 | [13] | **PST**:F330,W84 | | |
| 1AKU | FMN-A-150 | [14] | **HBO**:T59,W60 **HPI**:W60 **PST**:Y98 **WAT**:D63,Y100 | | |
| 1AY8 | PLP-A-413 | [15] | **HBO**:G108,T109,N194,S257 **PCT**:W140 **SBR**:K258,R266 | **HBO**:S255 | |
| 1BJU | GP6-A-910 | [16] | **HBO**:S190,S195 **HPI**:L99 **SPT**:H57 **WAT**:S190,V227 | | **HBO**:G219,D189 |
| 1BMA | 0QH-A-256 | [17] | **HBO**:S203, V224, Q200 **HPI**:F223, V103 | | |
| 1EVE | E20-A-2001 | [18] | **PCT**:F330 **PST**:W84,W279 | **PST**:W121 | |
| 1H2T | 7MG-Z-1152 | [18] | **HBO**:R112 **PST**:Y20,Tyr43 **SBR**:D116 | **HBO**:D114,R123,R127,Q133,V134 | |
| | | | | **SBR**:D114,R127 | |
| 1N7G | NDP-A-701 | [19] | **HBO**:T37,G38,Q39,D40,D92,S63,L92,A115,S117,Y128,Y185,K189,H215,R220 | **WAT**:I36 | **HBO**:D91 |
| | | | **PST**:R60 **SBR**:R60,R61,R69,R220 | | |
| | | | **WAT**:G35,T37,G38,D40,R60,R61,S63,N66,S117,Y128,K189,R220 | | |
| 1OSN | BVP-A-500 | [20] | **HBO**:Q90 **PST**:F93,F139 **SBR**:K25,R130 **WAT**:R143 | **HBO**:G22 | |
| 1P5E | TBS-A-301 | [21] | **HAL**:I10,L83 | | |
| 1VSN | NFT-A-283 | [22] | **HBO**:G66 | **HBO**:Q19,C25,D61,Y67 | |
| 1XDN | ATP-A-501 | [23] | **HBO**:I61,K87,V88,N92,R111 | | **HBO**:E86 |
| 2EFJ | 37T-A-502 | [24] | **HBO**:W161,S237 **PST**:Y157 | **PST**:Y368 | |
| 2IUZ | D1H-A-1440 | [25] | **HBO**:W137,W52,T138,W384 **PCT**:W137 **PST**:W52,W384 | | |
| | | | **WAT**:W52,W137,W384,T138 | **PST**:W137 | |
| 2REG | CHT-A-1 | [26] | **PCT**:W43,W90,W205,Y119 **SBR**:D45 | | |
| 2W0S | BVP-B-1207 | [27] | **HBO**:Y101,R72 **HAL**:N65 **HPI**:F68,Y101 **PST**:F68 **SBR**:R41,R93 | **SBR**:K17 | |
| 2ZOZ | ET-B-184 | [28] | **HPI**:L59,L88,W63,W113,F147 **PST**:W63,F147 | **PST**:W113,H153 | **HPI**:L100,V92,I152 |
| 3O1H | TMO-B-1 | [29] | **HBO**:W45 **PCT**:Y44 **WAT**:W45 | | |
| 3PXF | 2AN-A-305 | [30] | **HBO**:D145,F146 **HPI**:L55,K56 **SBR**:K33 | **HPI**:Y15,I35,V64,L66,L78 | |
| | 2AN-A-304 | [30] | **HPI**:I52,L76 **SBR**:K56,H71 | **HPY**:L78,V154,L37 | |
| 3R0T | FU9-A-338 | [31] | **HBO**:V116 **HPI**:V66,F113,I174 **PST**:H160 **SBR**:K68 | **HBO**:D175 **HPI**:L45,V53 | |
| | | | **WAT**:K68,W176 | **WAT**:D175 | |
| 3SHY | 5FO-A-1 | [32] | **HBO**:Q817 **HAL**:Y612 **HPI**:Y612 **PST**:F820 | **HPI**:V782,I813,M816,F820 | |
| 3TAH | BGO-A-300 | [33] | **HBO**:A11,K14,T15,S16,M114,A143,D113 **SBR**:D116 **WAT**:A11 | **HBO**:S12,G13,N113,L144 | |
| 3T5Y | MCS-B-116 | [34] | **HBO**:A327 **HPI**:R115 **SBR**:H295,R115 | **HBO**:N117,G118 | |
| 3THY | ADP-A-935 | [35] | **SBR**:K675 | | |
| 4ALW | HY7-A-1308 | [36] | **HBO**:D186 **SBR**:D186, E171 | | |
| 4KYA | 1UG-E-702 | [37] | **HBO**:A609 **HPI**:I402,L516,F520,M608 **PST**:W403,F520 **SBR**:D513 | **HBO**:Y553 | |
| 4PJT | 2YQ-D-1104 | [38] | **HBO**:G863 **PST**:Y889,Y907 | **HBO**:Q759,E763 | |
| 4QNB | 1B0-A-301 | [39] | **HBO**:N57,K70 **PCT**:K70 | | |
| 4RAO | 3L7-B-301 | [40] | **HBO**:R199,V187,K165,T141,K140,G139,T138,D137,R100,G69,K68 | | |
| | | | **PST**:F186 **WAT**:E133,D137,T141,M142,R199,G139 | | |
| 4RDL | FUC-A-604 | [41] | **HBO**:T347,G348,N395 **HPI**:W392 **WAT**:N395 | | |

# 7 References

## References

[1] Yang, J., Roy, A., and Zhang, Y. (Jan, 2013) BioLiP: a semi-manually curated database for biologically relevant ligand-protein interactions.. *Nucleic Acids Res,* **41**(Database issue), D1096–D1103.

[2] O'Boyle, N. M., Banck, M., James, C. a., Morley, C., Vandermeersch, T., and Hutchison, G. R. (January, 2011) Open Babel: An open chemical toolbox.. *Journal of Cheminformatics,* **3**(1), 33.

[3] Israelachvili, J. and Pashley, R. (1982) The hydrophobic interaction is long range, decaying exponentially with distance. *Nature,*.

[4] Waters, M. (2002) Aromatic interactions in model systems. *Current opinion in chemical biology,* pp. 736–741.

[5] Barril, X., Alemán, C., Orozco, M., and Luque, F. J. (July, 1998) Salt bridge interactions: stability of the ionic and neutral complexes in the gas phase, in solution, and in proteins.. *Proteins,* **32**(1), 67–79.

[6] McGaughey, G. B. (June, 1998) pi -Stacking Interactions. ALIVE AND WELL IN PROTEINS. *Journal of Biological Chemistry,* **273**(25), 15458–15463.

[7] Gallivan, J. P. and Dougherty, D. a. (August, 1999) Cation-pi interactions in structural biology.. *Proceedings of the National Academy of Sciences of the United States of America,* **96**(17), 9459–64.

[8] Barlow, D. J. and Thornton, J. M. (August, 1983) Ion-pairs in proteins.. *Journal of molecular biology,* **168**(4), 867–85.

[9] Jiang, L., Kuhlman, B., Kortemme, T., and Baker, D. (March, 2005) A "solvated rotamer" approach to modeling water-mediated hydrogen bonds at protein-protein interfaces.. *Proteins,* **58**(4), 893–904.

[10] Hubbard, R. and Haider, M. K. (2001) Hydrogen Bonds in Proteins: Role and Strength. *eLS,* **1**, 1–6.

[11] Auffinger, P., Hays, F. a., Westhof, E., and Ho, P. S. (November, 2004) Halogen bonds in biological molecules.. *Proceedings of the National Academy of Sciences of the United States of America,* **101**(48), 16789–94.

[12] Wilcken, R., Liu, X., Zimmermann, M. O., Rutherford, T. J., Fersht, A. R., Joerger, A. C., and Boeckler, F. M. (Apr, 2012) Halogen-enriched fragment libraries as leads for drug rescue of mutant p53.. *J Am Chem Soc,* **134**(15), 6810–6818.

[13] Harel, M., Schalk, I., Ehret-Sabatier, L., Bouet, F., Goeldner, M., Hirth, C., Axelsen, P. H., Silman, I., and Sussman, J. L. (Oct, 1993) Quaternary ligand binding to aromatic residues in the active-site gorge of acetylcholinesterase.. *Proc Natl Acad Sci U S A,* **90**(19), 9031–9035.

[14] McCarthy, A. A., Walsh, M. A., Verma, C. S., O'Connell, D. P., Reinhold, M., Yalloway, G. N., D'Arcy, D., Higgins, T. M., Voordouw, G., and Mayhew, S. G. (Sep, 2002) Crystallographic investigation of the role of aspartate 95 in the modulation of the redox potentials of Desulfovibrio vulgaris flavodoxin.. *Biochemistry,* **41**(36), 10950–10962.

[15] Okamoto, A., Nakai, Y., Hayashi, H., Hirotsu, K., and Kagamiyama, H. (Jul, 1998) Crystal structures of Paracoccus denitrificans aromatic amino acid aminotransferase: a substrate recognition site constructed by rearrangement of hydrogen bond network.. *J Mol Biol,* **280**(3), 443–461.

[16] Presnell, S. R., Patil, G. S., Mura, C., Jude, K. M., Conley, J. M., Bertrand, J. A., Kam, C. M., Powers, J. C., and Williams, L. D. (Dec, 1998) Oxyanion-mediated inhibition of serine proteases.. *Biochemistry,* **37**(48), 17068–17081.

[17] Peisach, E., Casebier, D., Gallion, S. L., Furth, P., Petsko, G. A., Hogan, Jr, J., and Ringe, D. (Jul, 1995) Interaction of a peptidomimetic aminimide inhibitor with elastase.. *Science,* **269**(5220), 66–69.

[18] Chakrabarti, P. and Bhattacharyya, R. (2007) Geometry of nonbonded interactions involving planar groups in proteins.. *Prog Biophys Mol Biol,* **95**(1-3), 83–137.

[19] Mulichak, A. M., Bonin, C. P., Reiter, W.-D., and Garavito, R. M. (Dec, 2002) Structure of the MUR1 GDP-mannose 4,6-dehydratase from Arabidopsis thaliana: implications for ligand binding and specificity.. *Biochemistry,* **41**(52), 15578–15589.

[20] Bird, L. E., Ren, J., Wright, A., Leslie, K. D., Degrève, B., Balzarini, J., and Stammers, D. K. (Jul, 2003) Crystal structure of varicella zoster virus thymidine kinase.. *J Biol Chem,* **278**(27), 24680–24687.

[21] De Moliner, E., Brown, N. R., and Johnson, L. N. (Aug, 2003) Alternative binding modes of an inhibitor to two different kinases.. *Eur J Biochem,* **270**(15), 3174–3181.

[22] Li, C. S., Deschenes, D., Desmarais, S., Falgueyret, J.-P., Gauthier, J. Y., Kimmel, D. B., Léger, S., Massé, F., McGrath, M. E., McKay, D. J., Percival, M. D., Riendeau, D., Rodan, S. B., Thérien, M., Truong, V.-L., Wesolowski, G., Zamboni, R., and Black, W. C. (Apr, 2006) Identification of a potent and selective non-basic cathepsin K inhibitor.. *Bioorg Med Chem Lett,* **16**(7), 1985–1989.

[23] Deng, J., Schnaufer, A., Salavati, R., Stuart, K. D., and Hol, W. G. J. (Oct, 2004) High resolution crystal structure of a key editosome enzyme from Trypanosoma brucei: RNA editing ligase 1.. *J Mol Biol,* **343**(3), 601–613.

[24] McCarthy, A. A. and McCarthy, J. G. (Jun, 2007) The structure of two N-methyltransferases from the caffeine biosynthetic pathway.. *Plant Physiol,* **144**(2), 879–889.

[25] Schüttelkopf, A. W., Andersen, O. A., Rao, F. V., Allwood, M., Lloyd, C., Eggleston, I. M., and van Aalten, D. M. F. (Sep, 2006) Screening-based discovery and structural dissection of a novel family 18 chitinase inhibitor.. *J Biol Chem,* **281**(37), 27278–27285.

[26] Oswald, C., Smits, S. H. J., Höing, M., Sohn-Bösser, L., Dupont, L., Le Rudulier, D., Schmitt, L., and Bremer, E. (Nov, 2008) Crystal structures of the choline/acetylcholine substrate-binding protein ChoX from Sinorhizobium meliloti in the liganded and unliganded-closed states.. *J Biol Chem,* **283**(47), 32848–32859.

[27] Caillat, C., Topalis, D., Agrofoglio, L. A., Pochet, S., Balzarini, J., Deville-Bonne, D., and Meyer, P. (Nov, 2008) Crystal structure of poxvirus thymidylate kinase: an unexpected dimerization has implications for antiviral therapy.. *Proc Natl Acad Sci U S A,* **105**(44), 16900–16905.

[28] Itou, H., Watanabe, N., Yao, M., Shirakihara, Y., and Tanaka, I. (Oct, 2010) Crystal structures of the multidrug binding repressor Corynebacteriumglutamicum CgmR in complex with inducers and with an operator.. *J Mol Biol,* **403**(2), 174–184.

[29] Moore, J. O. and Hendrickson, W. A. (Apr, 2012) An asymmetry-to-symmetry switch in signal transmission by the histidine kinase receptor for TMAO.. *Structure,* **20**(4), 729–741.

[30] Betzi, S., Alam, R., Martin, M., Lubbers, D. J., Han, H., Jakkaraj, S. R., Georg, G. I., and Schönbrunn, E. (May, 2011) Discovery of a potential allosteric ligand binding site in CDK2.. *ACS Chem Biol,* **6**(5), 492–501.

[31] Battistutta, R., Cozza, G., Pierre, F., Papinutto, E., Lolli, G., Sarno, S., O'Brien, S. E., Siddiqui-Jain, A., Haddach, M., Anderes, K., Ryckman, D. M., Meggio, F., and Pinna, L. A. (Oct, 2011) Unprecedented selectivity and structural determinants of a new class of protein kinase CK2 inhibitors in clinical trials for the treatment of cancer.. *Biochemistry,* **50**(39), 8478–8488.

[32] Xu, Z., Liu, Z., Chen, T., Chen, T., Wang, Z., Tian, G., Shi, J., Wang, X., Lu, Y., Yan, X., Wang, G., Jiang, H., Chen, K., Wang, S., Xu, Y., Shen, J., and Zhu, W. (Aug, 2011) Utilization of halogen bond in lead optimization: a case study of rational design of potent phosphodiesterase type 5 (PDE5) inhibitors.. *J Med Chem,* **54**(15), 5607–5611.

[33] Ash, M. R., Maher, M. J., Guss, J. M., and Jormakka, M. (Dec, 2011) The structure of an N11A mutant of the G-protein domain of FeoB.. *Acta Crystallogr Sect F Struct Biol Cryst Commun,* **67**(Pt 12), 1511–1515.

[34] Bretschneider, T., Zocher, G., Unger, M., Scherlach, K., Stehle, T., and Hertweck, C. (Feb, 2012) A ketosynthase homolog uses malonyl units to form esters in cervimycin biosynthesis.. *Nat Chem Biol,* **8**(2), 154–161.

[35] Gupta, S., Gellert, M., and Yang, W. (Jan, 2012) Mechanism of mismatch recognition revealed by human MutS$\beta$ bound to unpaired DNA loops.. *Nat Struct Mol Biol,* **19**(1), 72–78.

[36] Tsuhako, A. L., Brown, D. S., Koltun, E. S., Aay, N., Arcalas, A., Chan, V., Du, H., Engst, S., Franzini, M., Galan, A., Huang, P., Johnston, S., Kane, B., Kim, M. H., Laird, A. D., Lin, R., Mock, L., Ngan, I., Pack, M., Stott, G., Stout, T. J., Yu, P., Zaharia, C., Zhang, W., Zhou, P., Nuss, J. M., Kearney, P. C., and Xu, W. (Jun, 2012) The design, synthesis, and biological evaluation of PIM kinase inhibitors.. *Bioorg Med Chem Lett,* **22**(11), 3732–3738.

[37] Zaware, N., Sharma, H., Yang, J., Devambatla, R. K. V., Queener, S. F., Anderson, K. S., and Gangjee, A. (Oct, 2013) Discovery of potent and selective inhibitors of Toxoplasma gondii thymidylate synthase for opportunistic infections.. *ACS Med Chem Lett,* **4**(12), 1148–1151.

[38] Aoyagi-Scharber, M., Gardberg, A. S., Yip, B. K., Wang, B., Shen, Y., and Fitzpatrick, P. A. (Sep, 2014) Structural basis for the inhibition of poly(ADP-ribose) polymerases 1 and 2 by BMN 673, a potent inhibitor derived from dihydropyridophthalazinone.. *Acta Crystallogr F Struct Biol Commun,* **70**(Pt 9), 1143–1149.

[39] Bhattacharya, A., Alam, S. L., Fricke, T., Zadrozny, K., Sedzicki, J., Taylor, A. B., Demeler, B., Pornillos, O., Ganser-Pornillos, B. K., Diaz-Griffero, F., Ivanov, D. N., and Yeager, M. (Dec, 2014) Structural basis of HIV-1 capsid recognition by PF74 and CPSF6.. *Proc Natl Acad Sci U S A,* **111**(52), 18625–18630.

[40] Keough, D. T., Špaček, P., Hocková, D., Tichý, T., Vrbková, S., Slavětínská, L., Janeba, Z., Naesens, L., Edstein, M. D., Chavchich, M., Wang, T.-H., de Jersey, J., and Guddat, L. W. (Mar, 2013) Acyclic nucleoside phosphonates containing a second phosphonate group are potent inhibitors of 6-oxopurine phosphoribosyltransferases and have antimalarial activity.. *J Med Chem,* **56**(6), 2513–2526.

[41] Hao, N., Chen, Y., Xia, M., Tan, M., Liu, W., Guan, X., Jiang, X., Li, X., and Rao, Z. (Dec, 2014) Crystal structures of GI.8 Boxer virus P dimers in complex with HBGAs, a novel evolutionary path selected by the Lewis epitope.. *Protein Cell,*.
